# Adapting and Evaluating a Rapid, Low-Cost Method to Enumerate Flies in the Household Setting

**DOI:** 10.4269/ajtmh.16-0162

**Published:** 2017-02-08

**Authors:** Marlene K. Wolfe, Holly N. Dentz, Beryl Achando, MaryAnne Mureithi, Tim Wolfe, Clair Null, Amy J. Pickering

**Affiliations:** 1 Tufts University, Medford, Massachusetts.; 2 Innovations for Poverty Action, New Haven, Connecticut.; 3 Emory University, Atlanta, Georgia.; 4 University of California Davis, Davis, California.; 5 Mathematica Policy Research, Washington, District of Columbia.; 6 Stanford University, Stanford, California.

## Abstract

Diarrhea is a leading cause of death among children under 5 years of age worldwide. Flies are important vectors of diarrheal pathogens in settings lacking networked sanitation services. There is no standardized method for measuring fly density in households; many methods are cumbersome and unvalidated. We adapted a rapid, low-cost fly enumeration technique previously developed for industrial settings, the Scudder fly grill, for field use in household settings. We evaluated its performance in comparison to a sticky tape fly trapping method at latrine and food preparation areas among households in rural Kenya. The grill method was more sensitive; it detected the presence of any flies at 80% (433/543) of sampling locations versus 64% (348/543) of locations by the sticky tape. We found poor concordance between the two methods, suggesting that standardizing protocols is important for comparison of fly densities between studies. Fly species identification was feasible with both methods; however, the sticky tape trap allowed for more nuanced identification. Both methods detected a greater presence of bottle flies near latrines compared with food preparation areas (*P* < 0.01). The grill method detected more flies at the food preparation area compared with near the latrine (*P* = 0.014) while the sticky tape method detected no difference. We recommend the Scudder grill as a sensitive fly enumeration tool that is rapid and low cost to implement.

## Introduction

Diarrhea is a leading cause of morbidity and mortality among children in developing countries. Over 700,000 deaths worldwide were attributable to diarrheal disease in 2013,^1^ and 50% of deaths of children < 5 years from diarrhea occurred in sub-Saharan Africa in 2011.^1^ Most diarrheal pathogens are transmitted through the fecal–oral route. Interventions therefore focus on the separation of human waste from the environment through the provision of clean water, latrine improvement, and proper hygiene. However, flies are also an important, yet sometimes overlooked, vector for transmission of diarrheal-disease-causing pathogens within households.^2^ Intervention studies have demonstrated that reductions in fly activity due to trapping, spraying, or improved sanitation can reduce diarrhea by 22–42%.^3^–^5^

Flies land on feces, food, and household surfaces that humans are in contact with daily. Filth flies in the families Muscidae (houseflies), Sarcophagidae (flesh flies), and Calliphoridae (bottle flies) all thrive when living in close proximity to humans.^2^ These flies take advantage of breeding and feeding opportunities in human waste, especially when feces are not safely contained. Studies consistently show that these flies can carry microorganisms and deposit them with their mouth or legs when landing on surfaces.^6^–^8^ In one German study, researchers tested wild-caught flies from 12 species for pathogenic and nonpathogenic microorganisms. Every fly tested transmitted at least two species of bacteria when landing on blood agar plates.^9^ Many pathogens known to cause clinical diarrhea have been detected on flies, including enteropathogenic *Escherichia coli*, *Salmonella*, *Shigella*, *Cryptosporidium parvum*, and *Giardia lamblia*.^10^–^12^

There is no standardized method for measuring fly densities in the household setting. Methods and reported implementation details vary widely between studies, and researchers often do not clearly justify their methods.^13^,^14^ Most methods were developed to provide a tool to judge whether farms and other food producers met minimum hygiene requirements for their industry.^15^ These tools are now used in industrial settings as well as community health research (e.g., assessing the impact of fly control interventions in low-income settings). Measurement methods exploit fly behavior, such as an attraction to food or animal waste or the tendency to land on straight edges. Techniques reported in the literature include bucket traps baited with fly attractants, sticky tape or sticky paper traps, and counting flies that land on a specialized grill.^14^–^19^ The ideal fly enumeration method should be fast, inexpensive, and portable while providing an accurate measurement of fly density.

Baited traps use the attraction of flies to decaying material, such as decomposing waste or rotting meat, to recruit the maximum number of flies in an area. One disadvantage of this method is that the bait may attract a larger number of flies than would gather naturally; baits can also fail to outcompete other olfactory attractants in the area or differentially attract certain fly species.^19^ The type of bait must be carefully considered if the goal is to attract multiple relevant fly species.

Hanging sticky fly tape is a popular method to measure fly density without olfactory attractants. Sticky tape attracts flies visually by providing straight, light-colored surfaces for them to land on where flies are trapped and may be easily viewed and counted.^16^,^17^ However, field workers may find the process of setting the traps difficult and unpleasant because the tape is challenging to handle. It can often take more than 20 minutes to set one trap. Field workers may set traps inconsistently; the direction and tautness of the tape may affect fly capture. Field teams usually need to make a return trip to the measurement site to retrieve the tape to allow time for flies to collect, which increases the cost of assessment.

The fly grill technique, introduced by Scudder in 1947, allows the user to determine fly density in a given area by observing the number of flies that land on a grill during a set period.^18^ The grill usually consists of 16–24 slats measuring between 20 and 80 cm in length that are painted bright yellow and arranged to form a square. The bright surface and straight lines of the grill visually attract flies in the area; the grill is observed and the number of flies that land on the grill is counted over a period lasting 30–90 seconds. In industrial settings, the grill is usually placed in the area with the highest observed fly activity.^18^ The method is rapid (< 5 minutes), although it may be challenging to count flies quickly as they land since flies are not trapped and may land on the grill multiple times (this may be particularly difficult if the fly density is high). The technique is recommended by the U.S. Food and Drug Administration to quantify fly densities at poultry farms.^20^

Our study objective was to determine the feasibility and utility of the Scudder grill as a low-cost technique to rapidly assess the presence and density of flies in household environments. We also compared the performance of the grill to a sticky tape fly enumeration method. Additional objectives were to assess the relationship between fly presence and sanitation access, and to profile the fly species present at food preparation versus latrine areas in homes.

## Methods

### Setting and study population.

This study was conducted in households within Kakamega and Bungoma counties located in western Kenya, a region where extended families typically live together in compounds and most engage in subsistence agriculture. In Kakamega and Bungoma, 6% and 5% of households have access to electricity, respectively, and in both counties, 11% of adults have completed secondary education.^21^ Study households were selected from rural villages within these counties. The data used in this study were collected from a subset of households surveyed as part of the baseline assessment for a cluster-randomized controlled trial (the WASH benefits study), evaluating the child health impacts of water, sanitation, hygiene, and nutritional interventions.^22^ Households were selected to participate as part of the WASH benefits study if there was a pregnant woman in her second or third trimester.^22^ Data collection for this study of flies was conducted among a subset of households selected for an assessment of parasitic infection, defined as those enrolled households who had a child aged 18–27 months living in the family compound. Flies were enumerated at the latrine and food preparation areas of study compounds using the Scudder fly grill and a sticky fly tape method simultaneously. These areas within the compound were chosen as potentially important areas for fly-mediated enteric pathogen transmission.

### Scudder fly grill.

**Figure 1.**
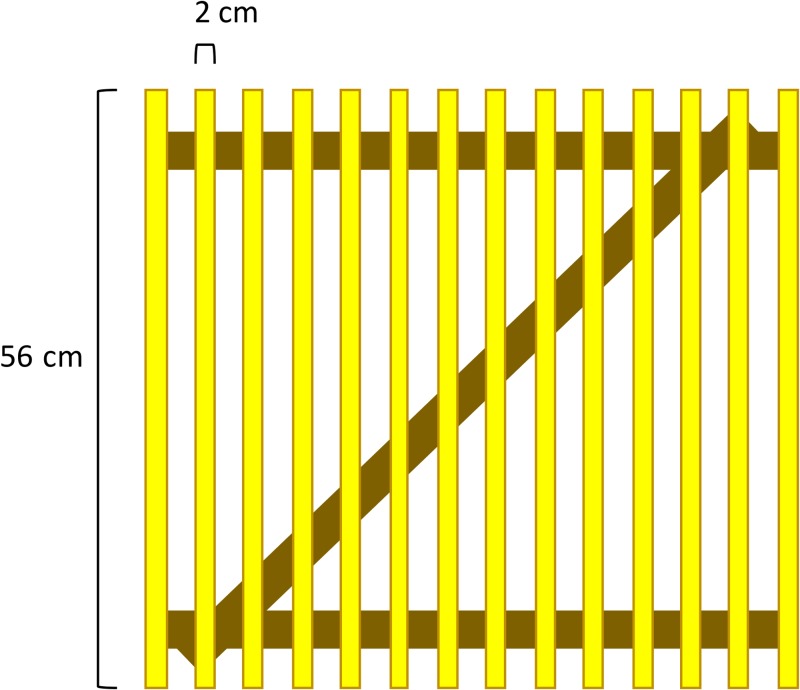
Scudder grill design. The grill measures 56 × 56 cm, with 2-cm-wide slats and 2 cm space in between slats.

Fly density was measured using a modified Scudder fly grill sized slightly smaller than the recommended range of sizes described by Scudder; the size was selected for portability in a field setting where it might need to be hand carried for long walking distances. The grill consisted of 14 parallel wooden slats 56 cm in length and each 2 cm wide, painted bright yellow, and supported by two perpendicular slats and one diagonal slat (Figure 1

). This size was chosen to balance portability with maximum surface area for fly landings.

### Sticky fly tape.

Revenge^®^ Sticky Fly Tape (Roxide International Inc, Larchmont, NY) was also used to measure fly density at the same households. Enumerators cut three strips of fly tape 1.5 feet in length and strung the tape strips horizontally and parallel to one another. Field staff used string to attach the tape to structural elements of the house or latrine (or to pins inserted in the walls); tape was pulled taut to create a straight line. Tape was hung inside the latrine and near the food preparation area. Enumerators were careful to hang tape away from smoke emitted by the cooking fire and ensure that tape was shielded from heavy rainfall. Enumerators coated their fingers in petroleum jelly before unrolling the tape to prevent it from sticking to their fingers and disrupting the adhesive. Tape was hung a minimum of 4 feet above the ground to keep it out of reach of children and animals.

### Fly enumeration.

Each participating household was visited for data collection on two consecutive days. On day 1, enumerators measured fly density at the most commonly used food preparation area as well as near the primary latrine using the grill method. The grill was placed on the ground in the doorway of the latrine, and placed as close as possible to cooking utensils in the food preparation area. Enumerators stood 1 m away from the grill and remained still while using a stopwatch to time counting periods. During a 90-second counting period, enumerators used a mechanical tally counter to record the number of flies landing on the grill. Enumerators also identified the fly species that landed on the grill by category, as described in the section, Fly species identification. After recording values for the first 90-second period, enumerators repeated the counting and fly species identification during a second 90-second observation period. Each fly landing was counted as one fly (because it was not feasible to distinguish one fly from another fly of the same species); thus, it is possible that flies landed on the grill and were counted multiple times.

Enumerators then hung fly tape at the same food preparation area and at the latrine. Enumerators recorded the weather at compounds on day 1. Enumerators rated the weather as hot, warm, or cold; the air as dry or moist; and the overall weather as sunny, partially sunny, or not sunny. Enumerators also noted if the grill was placed in the sun, partially in the sun, or not in the sun.

On the next day, enumerators returned to the same households to retrieve the sticky fly tape. The tape was removed, and the number of flies were recorded and the species were identified (identification details below). Enumerators then conducted an interview with a respondent in the household regarding sanitation access and behaviors among household members; they also observed the latrine and household area and recorded information about latrine construction, apparent use and condition, and the presence of human or animal feces in and around the household. Sticky tape containing the captured flies was transported to the field laboratory for safe disposal.

### Fly species identification.

Enumerators were trained over the course of a week to identify fly species when enumerating the flies. Enumerators were instructed to exclude any insects from counts that were not synanthropic flies (e.g., bees, wasps, ants, and fruit flies). After extensive piloting to assess the feasibility of real-time species identification during fly enumeration in the field, field staff were instructed to categorize flies into three main categories—houseflies (primarily *Musca domestica*, *Fannia canicularis*, *Stomoxys* spp.), bottle flies (primarily *Calliphora* spp., *Lucilia* spp., *Chrysomya* spp.), and flesh flies (primarily *Sarcophaga* spp.). When enumerating flies on sticky tape, houseflies were further categorized as *M. domestica*, *F. canicularis*, or *Stomoxys* spp. This additional specificity was possible because flies were immobilized and enumerators had more time to examine fly characteristics.

### Analysis.

The analysis was designed to cover a range of topics of possible interest to researchers intending to use these fly enumeration methods in the field. A comparison of the ability of the two methods to detect any fly activity was done using Cohen's kappa statistic, which measures agreement in the presence/absence of flies detected at each location. A χ^2^ test of association was used to assess the relationship between fly presence at each sampling site within the same household, and the Wilcoxon signed-rank test for matched data was used to assess the difference in density between sampling locations within a household for each method. For weather analysis we grouped “hot” and “warm” ratings together and used the Wilcoxon rank-sum test to assess the difference between hot/warm versus cool weather. We grouped “sun” and “partial sun” together to assess the difference between the presence of any sun and no sun on fly counts with the grill method. We also used the Wilcoxon rank-sum test to assess if evidence of tampering with the sticky tape affected results. We performed a natural log transformation of fly counts to more closely approximate a normal distribution for the purpose of regression modeling (adding 1 to all values to allow for transformation of 0 counts). Linear regression was used to assess the relationship between time of day that the grill count was taken and fly counts. We used data generated by both the grill and tape methods to assess the relationship between fly counts at the latrine area to latrine features and household characteristics using linear regression. Bivariate linear regressions were first performed for each variable of interest (Supplemental Table 1); variables associated with fly density with a *P* < 0.2 for either the grill or the tape were included in one multivariate model. Differences in species identification between methods and location were assessed using Pearson's χ^2^ test. *P* values less than 0.05 were considered to be statistically significant. *P* values were not adjusted for multiple testing. All fly density measurements included counts of 0. All statistical analysis was performed using Stata 13 (StataCorp LP, College Station, TX).

## Results

Fly density measurements were attempted at 646 households from June 2013 to May 2014. Data were collected for food preparation areas with the grill at 563 households (90% of those who consented to measurements) and with the sticky tape at 387 households (60%). Data for latrines were collected with the grill at 453 households (86%) and with the sticky tape at 230 households (36%). Of households where tape was not hung, 22% refused for tape to be hung and 73% had no structure from which to hang the tape. Some households sharing a neighbor's latrine allowed observation with the grill, but did not consent to hanging sticky tape. In some cases, tape was hung but field challenges such as weather or absence of household members for field visits meant that tape was not collected in a timely enough manner for data to be recorded. We did not observe a significant difference between households that were measured using both methods in comparison to those with just one (Supplemental Table 2). Field managers estimated grill measurements took less than 5 minutes, whereas tape hanging required 20 minutes.

When considering latrine characteristics, 66% (217/329) of households shared with other households; 20% (61/312) had a concrete slab and only 6% (20/312) had a ventilation pipe. When inspected, 99% (308/312) of latrines had a well-worn path suggesting regular use. Households had an average of two cows and 12 chickens living on the compound.

**Figure 2.**
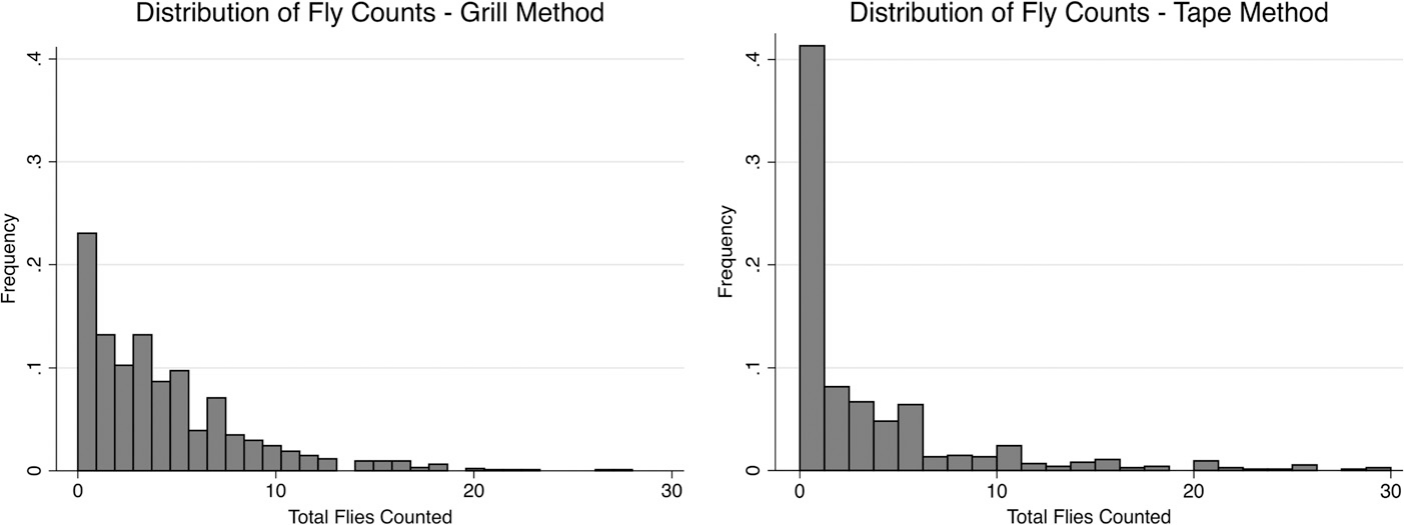
Frequency of flies counted at both sites using the grill and tape methods. Three outliers were excluded from grill data, and 16 were excluded from tape data in figures only (all data were included in statistical analysis).

We found a median of three flies (mean = 4.5, range = 0–89) at the household food preparation area using the Scudder grill method and a median of one fly (mean = 5.2, range = 0–200) using the sticky tape method. At the latrine area, we found a median of three flies using the Scudder grill (mean = 3.9 flies, range = 0–37) and a median of two flies using sticky tape (mean = 5.2 flies, range = 0–72) (Figure 2

). Considering only positive counts, the median number of flies enumerated by the grill was four flies (*N* = 446) at the food area and four flies (*N* = 352) at the latrine area; the median number of flies captured by the tape was four flies (*N* = 224) at the food area and three flies (*N* = 166) at the latrine area.

### Sensitivity.

At the food preparation area, there was slight agreement between the ability of the two methods to detect fly presence (κ = 0.063, 95% confidence interval [CI] = −0.034–0.160), and at the latrine there was slight to no agreement between the two methods (κ = −0.072, 95% CI = −0.195–0.051). Supplemental Figure 1 shows the Bland–Altman plot comparing measurements made by the two methods. The grill method detected fly presence at a greater percentage of households than the tape method at both sampling sites (Table 1). At households that had data for both methods at a given location, flies were counted at 59% of food preparation areas using the tape method, whereas the grill method detected flies at 79% of food preparation areas. Similarly, 73% of household had flies present at the latrine according to tape enumeration data, compared with 81% of latrines according to grill enumeration data.

### Fly activity in food preparation area compared with latrine area.

We used data from each method to compare the presence of fly activity in the latrine area to the presence of fly activity in the food preparation area. Fly presence at the food preparation area was significantly and positively associated with the presence of flies at the latrine within the same household (grill method χ^2^ = 42.36, *P* < 0.001; tape method χ^2^ = 15.79, *P* < 0.001). We also found that fly density was significantly higher at the food preparation area than the latrine area by the grill method, although the medians were similar (median 3 versus 3, mean 4.50 versus 3.93; *z* = −2.464, *P* = 0.014, *N* = 450); data from the tape method indicated no significant difference in the number of flies between the two areas (median 2 versus 1, mean 5.24 versus 5.24; *z* = 1.540, *P* = 0.124, *N* = 220).

### Weather.

The grill method detected significantly more flies on days that enumerators described as warm or hot compared with those described as cold at both the food area and the latrine area (*P* = 0.001 at both locations; Table 2). At both the latrine and kitchen areas, the grill detected significantly more flies on sunny days (*P* < 0.01); the grill also detected more flies when its placement was in full sunlight (*P* = 0.028). However, whether the air was observed as dry or humid (moist), had no significant effect on the number of flies detected by the grill at either location (*P* = 0.395). Neither temperature (*P* = 0.315), sun (*P* = 0.621), nor humidity (*P* = 0.263) had a significant association with the number of flies found using the tape method.

### Logistical considerations.

According to the protocol, fly tape was hung for 24 hours before it was removed and flies counted. Reaching households in rural areas exactly 24 hours after the initial hanging was consistently difficult. As a result, the actual time that fly tape was hanging ranged from 23.6 to 32.9 hours at the latrine and from 24.9 to 33.1 hours at the food preparation area. However, we found the number of hours that the fly tape was hanging was not significantly associated with the number of flies counted (latrine area *P* = 0.660, food area *P* = 0.409; Supplemental Table 3).

We found that the tape was vulnerable to tampering by household members or animals. Tape at the latrine was damaged or tampered with at 6.4% of households while 8.2% of households had tape damaged at the food preparation area. Evidence of tampering did not result in significantly different fly counts from houses where it remained intact at the food preparation (*z* = −0.219, *P* = 0.8267) or latrine (*z* = 0.792, *P* = 0.4283) areas. We found no relationship between the time of day that fly counts were taken with the grill (in minutes after midnight) and the number of flies observed (latrine area *P* = 0.866; food area *P* = 0.233; Supplemental Table 3).

### Relationship of fly density to latrine characteristics.

Using the grill data, we found that there was a significant association between the presence of a roof on the latrine and the number of flies counted, with fewer flies associated with a roof (median with roof = 1, median without roof = 3, *P* = 0.015; Table 3). According to tape enumeration data, we found a significant association between and decrease in flies captured on tape and latrine sharing (median shared = 3, median not shared = 2, *P* = 0.008).

### Fly species profiles.

**Figure 3.**
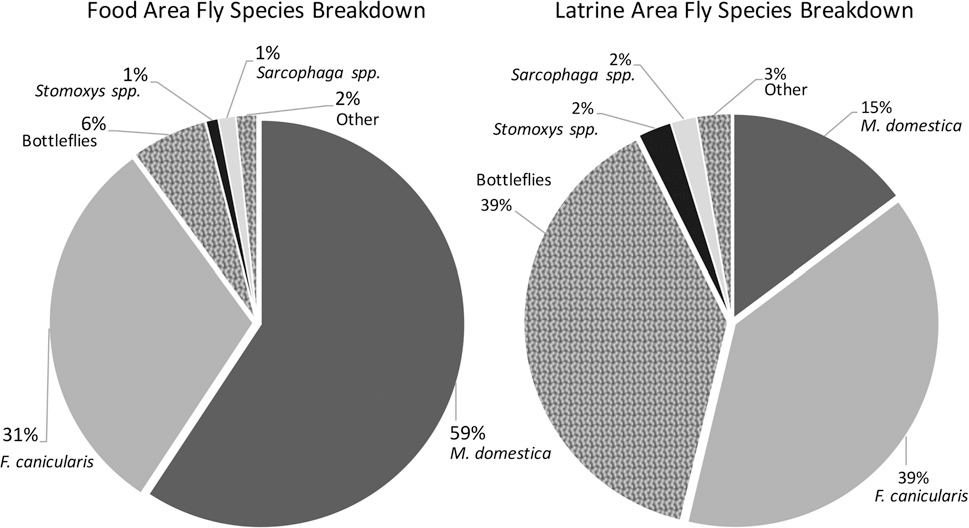
Percentage of fly species identified at each area measured by the sticky tape method.

For each fly species category, neither method detected flies of that species at a greater proportion of households at each site (Table 4). However, at 11% of food areas and 24% of latrines, the species of flies observed using the grill method could not be identified due to limited observation time. The grill method detected houseflies at a larger proportion of food areas than latrine areas (*P* = 0.002) but did not detect a difference in proportions of bottle or flesh flies at latrine areas (Supplemental Figure 2). The tape method did not detect bottle or flesh flies at a significantly different proportion of food versus latrine areas, but did detect more houseflies at the food area (*P* = 0.003). When we compared the total number of flies of each fly category counted on the tape (Figure 3

), we found that 39% of flies counted at the latrine were bottle flies, but only 6% of flies in the food preparation area were bottle flies. Species of housefly also varied between the two sites. At the latrine area, 15% of flies were *M. domestica* and 39% were *F. canicularis*, compared with 59% *M. domestica* and 31% *F. canicularis* at the food preparation area.

## Discussion

This study presents advantages and disadvantages of two methods for measuring fly densities under field conditions in a low-resource setting. We found that both methods were useful for fly enumeration; however, the grill had several clear advantages over the use of sticky tape. First, the grill had higher sensitivity; 79% of households had fly activity according to the grill compared with 59% by sticky tape. These results suggest the 3-minute measurement window is sufficient to capture fly activity with the grill. The time of day was not associated with grill fly counts, suggesting that timing of data collection does not strongly influence results. Second, the grill required far less time to implement in the field; only 5 minutes of field staff time was needed per observation compared with an implementation time of 45–60 minutes over two household visits 24 hours apart for the sticky tape. Third, the grill resulted in fewer missing data than the sticky tape (e.g., 14% versus 64% expected observations missing at the latrine area).

Fly density measurements with the grill were more responsive to weather conditions than tape measurements. Fly densities captured by the grill were higher on sunny and warm days. Previous studies have documented increased fly numbers during warm weather conditions.^6^,^23^,^24^ The responsiveness of the grill results to weather conditions suggests it is capturing a source of expected variation in fly density, but it could also indicate less accurate data if weather conditions are highly variable during the course of a day. The grill may be difficult to use in high fly density settings in which multiple flies are landing on the grill simultaneously.

The sticky tape method may be a good option when fly species need to be carefully identified and sufficient resources are available. This method is labor intensive and more difficult for enumerators to implement consistently. However, we found that slight variation in length of tape hanging or disruption of tape did not have a significant effect on the number of flies collected. We found no association between the warmth or humidity of a given day on the number of flies counted on tape, although this may be due to our reliance on enumerator reported weather conditions at one time point.

We were able to use results from both the tape data and the grill data to demonstrate significant relationships between fly density and latrine characteristics. Lower fly density was associated with a roof over the latrine, and higher fly density was associated with latrine sharing. A recent study using a bucket trap to measure fly density in pit latrines in Tanzania also found that the absence of a roof over the latrine was significantly associated with increased fly density in the latrine area; this was the only significant association they reported with latrine characteristics.^25^

Identifying the type of flies residing in households may help illuminate patterns of disease transmission, and standardized fly species identification methods could help generate evidence in this growing area of research. One disadvantage of the grill is that field staff have limited time to observe flies for species identification (11–24% of sites had flies that were not able to be identified); however, in this study we demonstrated that broad species classification was indeed feasible using the grill. We found that field workers were able to effectively sort flies into three main categories, and the species profile generated by this categorization matched both the results from the tape method and existing knowledge about fly species distribution and behavior.

*Chrysomya putoria* is a bottle fly known to breed in latrines in Africa, and other bottle fly species, such as *Lucilia* spp., are often found near human feces. In this study, we frequently detected bottle flies in the latrine area and observed some crossover into the food preparation area.^26^ Although it has been previously recognized that the houseflies can transmit human pathogens, *Fannia* spp. (lesser housefly) could present a different disease risk than *M. domestica* (greater housefly), because they have a greater attraction to feces than to human food.^26^ Recent studies have established that bottle flies, such as *C. putoria*, are likely to carry enteric pathogens.^27^ The oriental latrine fly, *Chrysomya megacephala* is similar to *Chrysomya* spp. found in east Africa, and studies have shown that it is significantly more likely to carry pathogens (including helminth eggs) than *M. domestica*^28^; another study found bottle flies were twice as likely to carry enteric bacteria than house or flesh flies.^29^ Species identification during fly enumeration could be increasingly valuable for assessing disease transmission risk.

Our results suggest that both tape and grill methods are useful for fly enumeration at households; however, the grill method is an especially cost-effective way of quickly assessing fly density and has increased sensitivity over the tape. When detailed species identification is not needed, we recommend the Scudder grill as a rapid detection tool for field studies. It should be noted that our investigation revealed poor concordance between the two methods evaluated. To best contribute to a body of literature that advances knowledge about the impact of flies on disease transmission in households, it would be ideal for studies to adopt a standardized fly enumeration protocol to facilitate direct comparison across studies.

## Supplementary Material

Supplemental figures and tables.

## Figures and Tables

**Table 1:** Fly presence using the grill versus tape methods

	Food preparation area			Latrine area		
	Tape (−)	Tape (+)	Total	Tape (−)	Tape (+)	Total
Grill (−)	10% (34)	11% (37)	21% (71)	4% (8)	15% (31)	19% (39)
Grill (+)	31% (104)	48% (160)	79% (264)	24% (49)	58% (120)	81% (169)
Total	41% (138)	59% (197)	100% (335)	27% (57)	73% (151)	100% (208)

The numbers in parenthesis represent the count of households in each cell.

**Table 2:** Fly counts (by grill and tape methods) and association with weather (Wilcoxon rank-sum test results)

	Food area						Latrine					
	Yes		No		Test stat	*P* value	Yes		No		Test stat	*P* value
	*n*	Median (IQR)	*n*	Median (IQR)			*n*	Median (IQR)	*n*	Median (IQR)		
Grill												
Sunny	512	3 (1–7)	50	2 (0–5)	−2.668	0.008*	415	3 (1–6)	37	1 (0–3)	−3.593	< 0.001*
Warm	519	3 (1–7)	43	2 (0–5)	−2.230	0.026*	417	3 (1–6)	35	0 (0–5)	−3.371	0.001*
Humid	273	3 (1–7)	289	3 (1–6)	−1.226	0.220	222	3 (1–6)	230	3 (0–5)	−0.851	0.395
Grill placed in sun	69	4 (2–7)	494	3 (1–6)	−2.238	0.025*	350	3 (1–6)	101	2 (0–5)	−2.203	0.028*
Tape												
Sunny	281	1 (0–4)	28	2 (0–5)	0.872	0.383	177	2 (0–6)	15	4 (0–10)	0.495	0.621
Warm	285	1 (0–5)	24	0 (0–3)	−1.531	0.126	179	3 (0–6)	13	0 (0–6)	−1.005	0.315
Humid	140	2 (0–5)	169	1 (0–4)	−1.286	0.198	90	3 (1–8)	102	2 (0–6)	−1.121	0.263

IQR = interquartile range.

* *P* < 0.05.

**Table 3:** Association between latrine characteristics and fly density: univariate and multivariate linear regression, with log-transformed fly density as dependent variable (*N* = 190)

	Grill				Tape			
	Univariate		Multivariate		Univariate		Multivariate	
	β	95% confidence interval	β	95% confidence interval	β	95% confidence interval	β	95% confidence interval
Stool on slab or floor	0.139	−0.032	0.123	−0.054	0.168	−0.120	0.073	−0.227
		0.310		0.300		0.457		0.373
Roof over the toilet	0.316*	0.039	0.321*	0.041	0.329	−0.471	0.339	−0.452
		0.594		0.602		1.130		1.129
Latrine shared with other households?	−0.030	−0.205	−0.036	−0.211	0.389*	0.097	0.389*	0.092
		0.145		0.139		0.681		0.686
Toilet has a slab	0.182	−0.034	0.166	−0.050	−0.277	−0.626	−0.279	−0.630
		0.397		0.381		0.072		0.071
Odor of feces	0.187	−0.068	0.121	−0.219	0.320	−0.139	−0.017	−0.602
		0.443		0.462		0.779		0.567
Odor of urine	0.142	−0.112	0.040	−0.292	0.381	−0.047	0.345	−0.182
		0.396		0.372		0.807		0.871
Drop hole is covered	−0.792	−1.770	−0.809	−1.784	0.116	−1.365	0.546	−0.935
		0.187		0.166		1.597		2.027

* *P* < 0.05.

**Table 4:** Fly species observed using grill and tape methods

	Food preparation area (*N* = 343)		Latrine area (*N* = 281)		Comparison of methods		Comparison of location	
	Grill	Tape	Grill	Tape	Food area (*P* value)	Latrine (*P* value)	Tape (*P* value)	Grill (*P* value)
Houseflies	79% (272)*	56% (193)*	49% (137)	42% (118)	0.289	0.259	0.003	0.002
Bottle flies	1% (22)	0% (6)	58% (163)	27% (75)	0.518	0.133	0.502	0.414
Flesh flies	0% (9)	0% (4)	15% (43)	4% (11)	0.741	0.787	0.712	0.128
Could not observe	11% (39)	–	24% (67)	–				

Note that households may have more than one type of fly, and counts are not mutually exclusive, *P* values from a Pearson's χ^2^ test. The numbers in parenthesis represent the count of households in each cell.

* Significantly more households with flies at this site compared with the other site, using the same measurement method (*P* < 0.05).
