# Supplementary material for: Adapting and Evaluating a Rapid, Low-Cost Method to Enumerate Flies in the Household Setting
Source: Am J Trop Med Hyg. 2017 Feb 8;96(2):449–56. doi: 10.4269/ajtmh.16-0162 (PMC5303052; doi:10.4269/ajtmh.16-0162)
Supplement: Supplementary file 1 [file SD10.pdf]

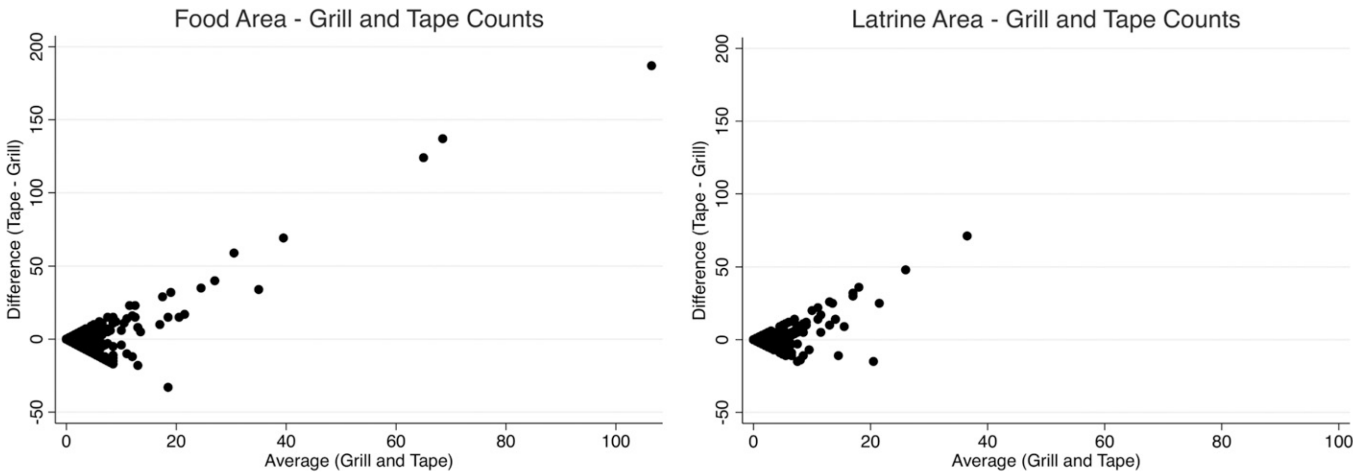

Supplemental Figure 1. Bland–Altman plots of the tape vs. the grill method, by sampling location.

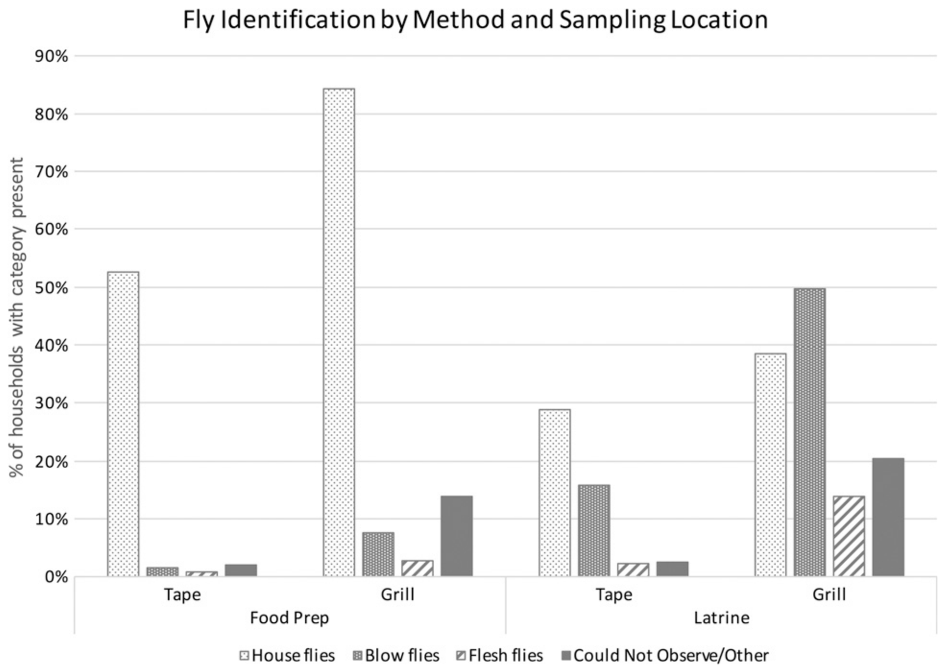

Supplemental Figure 2. Percentage of flies of each category identified by sampling location and enumeration method.

Supplemental Table 1

Association between latrine characteristics and fly density: bivariate linear regression with log-transformed fly density as dependent variable (*N* = 190)

	Grill				Tape			
	β	95% confidence interval		*P* value	β	95% confidence interval		*P* value
Latrine shared with other households?	0.030	–0.145	0.205	0.736	0.389	0.097	0.681	0.009
Stool on slab or floor	–0.139	–0.310	0.032	0.111	0.168	–0.120	0.457	0.252
Roof over the toilet	–0.316	–0.594	–0.039	0.026	0.329	–0.471	1.130	0.418
Toilet has a slab	–0.182	–0.397	0.034	0.098	–0.277	–0.626	0.072	0.119

Supplemental Table 2

Difference between mean fly density at households with both methods successfully used for measurement and those with only one method used

Measurement type	Location	Geo-mean flies (both measurement types)	Mean flies (single measurement type)	*P* value
Tape	Latrine	1.52	1.32	0.43
	Food preparation area	1.30	1.32	0.87
Grill	Latrine	1.51	1.50	0.86
	Food preparation area	1.59	1.62	0.69

Supplemental Table 3

Association of variability in fly tape hanging time and time of day of grill measurement with flies counted (linear regression results)

Dependent variable	Independent variable	β (SE)	*P* value	R^2
Log-mean fly count at latrine	Time tape was hanging (hours)	–0.05 (0.11)	0.660	0.0036
Log-mean fly count at food preparation area	Time tape was hanging (hours)	0.06 (0.07)	0.409	0.0095
Log-mean fly count at latrine	Time of day (minutes past midnight)	0.00 (0.00)	0.866	0.0001
Log-mean fly count at food preparation area	Time of day (minutes past midnight)	0.00 (0.00)	0.223	0.0026

SE = standard error.
